# 

**Published:** 1868-09-15

**Authors:** 


### CONTENTS:

ORIGINAL PAPERS:

Pathogenesis. The Pathological Anatomy and Pathogenesis of Disseminated Chronic Pneumonia, and of Pulmonary Tubercles. Translated by Walter Hay, M.D., Associate Editor, Chicago.. 583

Strangulated Inguinal Hernia at nine weeks of age. By Curtis J. Fenn, M.D., Chicago ........ 597

A Completed History........ 599

FOREIGN CORRESPONDENCE:

Letter from Samuel J. Jones, M.D ........ 606

PHILADELPHIA CORRESPONDENCE:

Letter from E. K. Hutchins........ 612

EDITORIAL:

Delay in Publication........ 613

Atomizing Apparatus........ 613

U. S. Marine Hospital........ 613

Honorary Degrees........ 613

BOOKS RECEIVED........ 614

CLINICAL LECTURES ON DISEASES OF THE EYE AND EAR, ETC........ 614

